# Granular Cell Tumor of the Breast Mimicking Carcinoma: A Case Highlighting Radiologic-Pathologic Discordance

**DOI:** 10.7759/cureus.107656

**Published:** 2026-04-24

**Authors:** Despoina Milonaki, Ioannis Provatas, Panagiota Pantoula, Argyro Fourlopoulou, Helen Trihia

**Affiliations:** 1 General Surgery, General Hospital of Nikaia "Agios Panteleimon", Athens, GRC; 2 Pathology, General Hospital of Nikaia "Agios Panteleimon", Athens, GRC; 3 Public Health Policy, University of West Attica, Athens, GRC; 4 Pathology, Metaxa Cancer Hospital Piraeus, Athens, GRC

**Keywords:** benign breast tumor, breast cancer, granular cell tumor of breast, radiologic-pathologic discordance, superparamagnetic iron oxide nanoparticles (spions)

## Abstract

Granular cell tumor (GCT) of the breast is a rare benign neoplasm of Schwann cell origin that can closely mimic breast carcinoma both clinically and radiologically, posing a significant diagnostic challenge. Core needle biopsy plays a crucial role in establishing the diagnosis. We present a case of a 72-year-old woman with a palpable right breast mass. Imaging findings were highly suspicious for malignancy, including high density on mammography and marked posterior acoustic shadowing on ultrasound. Initial core needle biopsy results were discordant with the radiological features, prompting repeat tissue sampling.

The patient underwent surgical excision following preoperative injection of superparamagnetic iron oxide (SPIO) nanoparticles (Magtrace) in anticipation of a potential sentinel lymph node biopsy. Intraoperative frozen-section analysis revealed a benign lesion, allowing avoidance of sentinel lymph node biopsy. Final histopathological and immunohistochemical evaluation demonstrated polygonal cells with abundant eosinophilic granular cytoplasm and positivity for S100 and CD68, consistent with a granular cell tumor. This case highlights the importance of radiologic-pathologic correlation in breast lesions with discordant findings and underscores the role of emerging techniques, such as SPIO tracers, in enabling flexible, patient-tailored surgical management, ultimately helping to avoid overtreatment.

## Introduction

Granular cell tumors (GCTs), also known as Abrikossoff tumors, are uncommon soft tissue neoplasms. They were first described by the Russian pathologist Abrikossoff in 1926 and were initially termed granular cell myoblastomas, as they were thought to be of muscular origin [1]. With advances in electron microscopy and immunohistochemistry, these tumors are now believed to originate from Schwann cells [2].

GCTs most commonly arise in the head and neck region [3]. Breast involvement is rare, accounting for approximately 5-15% of GCT cases [4], while it is less than 0.1% of all breast tumors [2,5]. They predominantly affect women in the fourth to sixth decades of life, although a wide age distribution has been reported [5,6]. A higher prevalence has been observed among individuals of African descent [6].

These tumors are usually solitary, with multifocal lesions described in approximately 10-25% of cases [7]. Breast GCTs are most commonly located in the upper inner quadrant, likely reflecting their origin from the distribution of supraclavicular nerves [5]. The vast majority are benign, whereas malignant variants are rare, accounting for approximately 1-2% of cases [2,7].

The clinical presentation of multiple GCTs has been associated with syndromes such as Noonan syndrome, neurofibromatosis type I, and LEOPARD syndrome. Mutations in the PTPN11 gene have also been reported in GCTs associated with these syndromes [8]. Clinically and radiologically, GCTs can mimic breast carcinoma [9]. Imaging findings are often non-specific and may resemble those of primary breast malignancy, posing a significant diagnostic challenge. While some lesions appear as well-circumscribed, rounded nodules, a considerable number exhibit irregular or stellate configurations. Additional mammographic findings may include spiculated margins, with or without calcifications, as well as secondary changes such as skin thickening or apparent extension toward the pectoralis muscle.

On ultrasound, these tumors are typically solid and heterogeneous, frequently displaying poorly defined margins. Imaging findings such as spiculated margins and posterior acoustic shadowing often raise suspicion for malignancy [10]. Histologically, GCTs consist of polygonal cells with eosinophilic granular cytoplasm [11]. Immunohistochemically, they typically express S100, CD68, and inhibin, while lacking cytokeratin expression [12]. Most tumors are benign, although rare malignant cases have been described [13].

According to the Fanburg-Smith criteria, GCTs are classified based on the presence of the following six histopathological features: necrosis, spindling of tumor cells, vesicular nuclei with prominent nucleoli, increased mitotic activity (>2 mitoses per 10 high-power fields), high nuclear-to-cytoplasmic ratio, and nuclear pleomorphism. Based on these criteria, tumors are categorized as benign, atypical, or malignant [14].

Local surgical excision remains the treatment of choice, with clear margins, given the low present risk of local recurrence, which is uncommon [15,16]. In the context of diagnostic uncertainty, particularly when lesions mimic breast carcinoma, accurate lymphatic staging may occasionally be considered. Sentinel lymph node biopsy (SLNB) has traditionally relied on radioactive tracers such as technetium-99m. However, superparamagnetic iron oxide (SPIO) tracers have emerged as a radiation-free alternative, offering increased flexibility in clinical practice. Notably, SPIO tracers can be administered well in advance of surgery and remain detectable within lymphatic tissue for prolonged periods, even beyond one month [17].

These considerations highlight the importance of optimizing diagnostic and surgical approaches in cases where clinical and radiological findings may be misleading. In this context, we present a case of breast granular cell tumor mimicking malignancy, emphasizing the radiologic-pathologic discordance and the implications for appropriate surgical management.

## Case presentation

**Figure 1:**
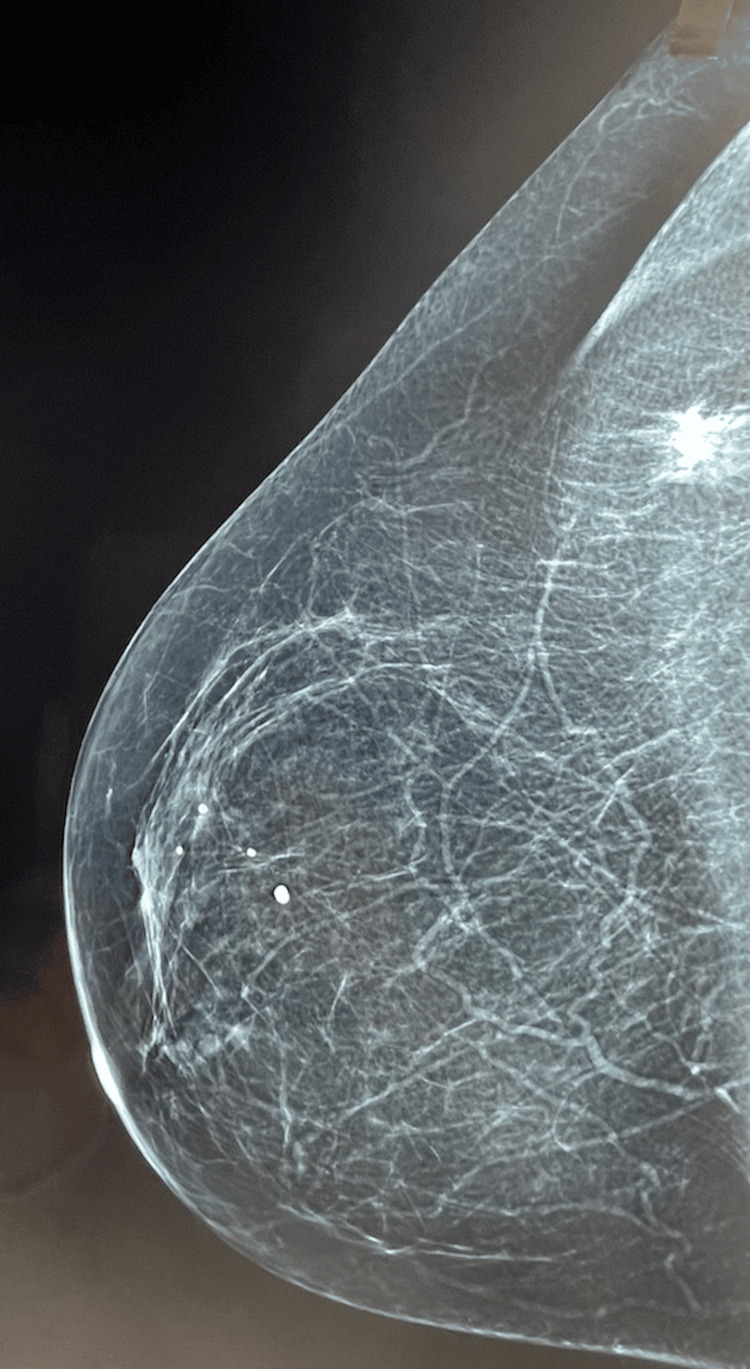
Right breast mammography depicting a high-density, irregular mass with spiculated margins.

**Figure 2:**
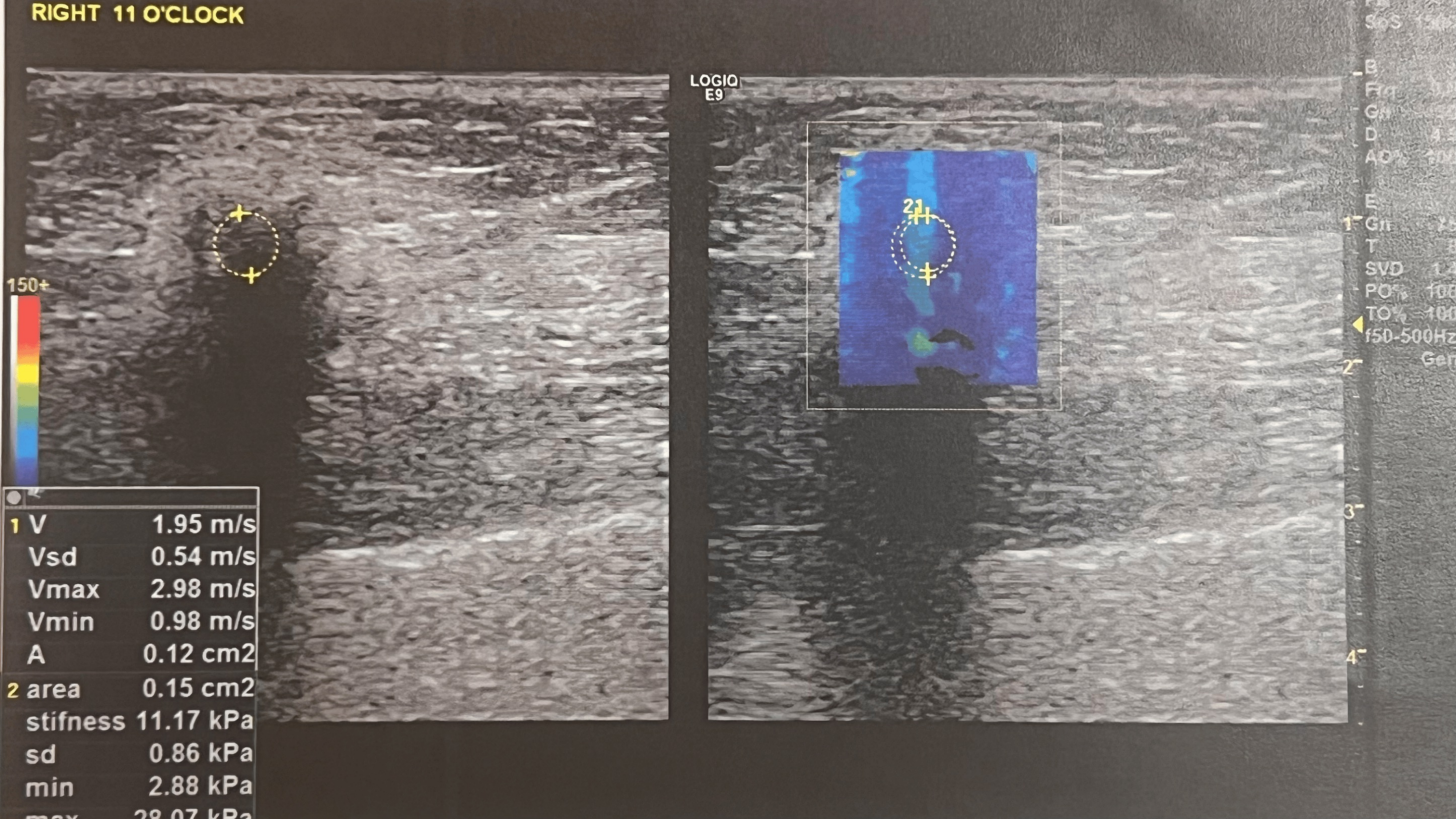
Right breast ultrasound depicting a hypoechoic lesion with posterior acoustic shadowing.

A 72-year-old woman presented in September 2024 with a palpable mass in the upper outer quadrant of the right breast. Her personal and family history was unremarkable. On clinical examination, a firm 2 cm mass was identified. Mammography revealed a high-density, irregular mass with spiculated margins (BI-RADS 4) (Figure 1), while hand-held breast ultrasound demonstrated a hypoechoic lesion with posterior acoustic shadowing (Figure 2).

Contrast-enhanced breast magnetic resonance imaging (MRI) was recommended; however, the patient refused due to claustrophobia. Initial core biopsy revealed breast tissue with non-specific inflammatory infiltrates, without features indicative of a neoplastic process. Due to radiologic-pathologic discordance, a repeat biopsy was performed; however, it yielded findings similar to those of the initial biopsy. Therefore, surgical excision was decided. One day prior to surgery, SPIO tracers (Magtrace) were injected [17]. Intraoperative frozen section revealed no evidence of malignancy, and sentinel lymph node biopsy was avoided.

Final histopathology confirmed a breast tissue specimen weighing 69 g and measuring 8 x 8.5 x 4 cm, covered by an elliptical skin segment measuring 5 x 2 cm. On sectioning, at a distance of 4 cm from the deep surgical margin and 2.5 cm from the skin, in an area marked with a surgical suture, there was a whitish, relatively irregularly shaped neoplastic lesion (entirely submitted in paraffin blocks) with a maximum diameter of 2.2 cm, of solid appearance and increased consistency on cut surface. In the remaining areas, the breast parenchyma shows no significant macroscopic alterations and contains two lymph nodes measuring 0.3 cm and 0.8 cm in greatest dimension.

Adipose tissue specimen measuring 5.6 x 4 x 1.5 cm, marked with a double suture. On sectioning and during frozen section examination, one lymph node measuring 1.1 cm in greatest dimension was identified. Subsequently, on routine gross examination, an additional six lymph nodes were identified, ranging from 0.1 to 0.4 cm in greatest dimension (entire specimen submitted). The frozen section examination of the above-described lymph node was negative for malignancy. Microscopically, a benign mesenchymal neoplasm of the breast (right breast) is identified, measuring 2.2 cm in greatest dimension.

**Figure 3:**
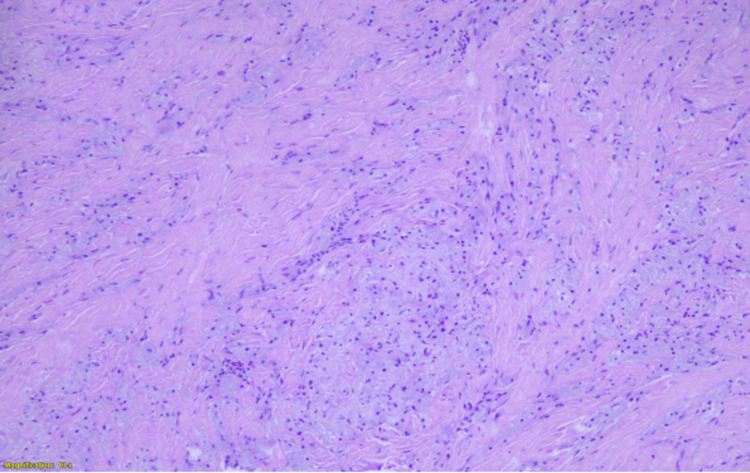
Benign neoplastic lesion arising from Schwann cells, composed of solid nests or trabecular arrangements (hematoxylin and eosin stain, ×4).

**Figure 4:**
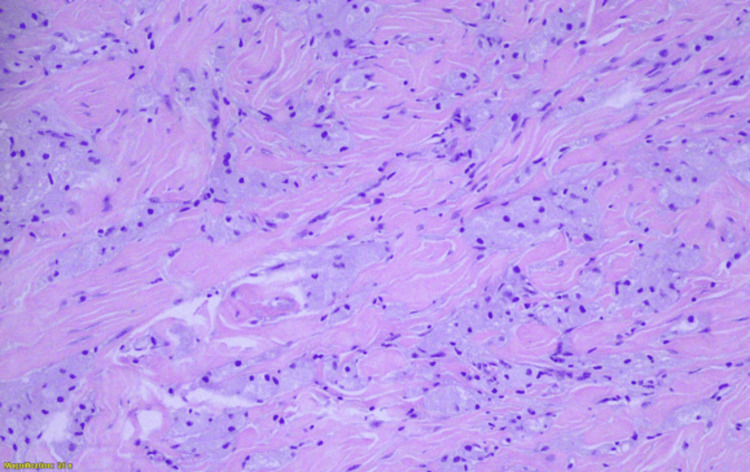
Polygonal or rounded, relatively large cells with eosinophilic, granular cytoplasm and small, rounded, uniform, hyperchromatic nuclei, without mitotic activity (hematoxylin and eosin stain, ×10).

More specifically, this was a benign neoplastic lesion of neuroectodermal origin, arising from Schwann cells of the perineurium, composed of solid nests and, to a lesser extent, trabecular arrangements of polygonal or rounded, relatively large cells, with eosinophilic, granular cytoplasm and small, rounded, uniform, hyperchromatic nuclei (Figure 3). No mitotic activity, spindle cell morphology, necrosis, vesicular nuclei with nucleoli, increased nuclear-to-cytoplasmic ratio, or nuclear pleomorphism were observed (Figure 4).

**Figure 5:**
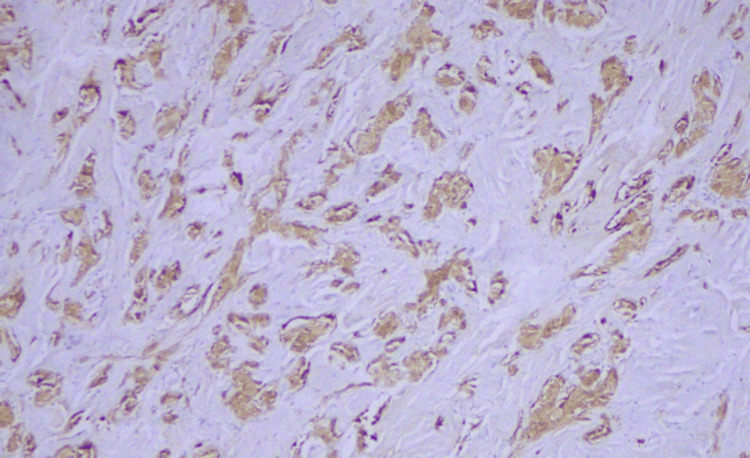
Immunohistochemistry: S-100 positivity.

**Figure 6:**
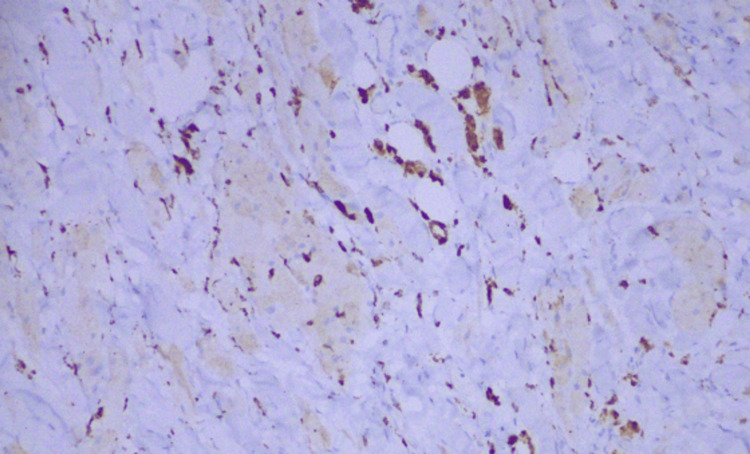
Immunohistochemistry: CD68 positivity.

**Figure 7:**
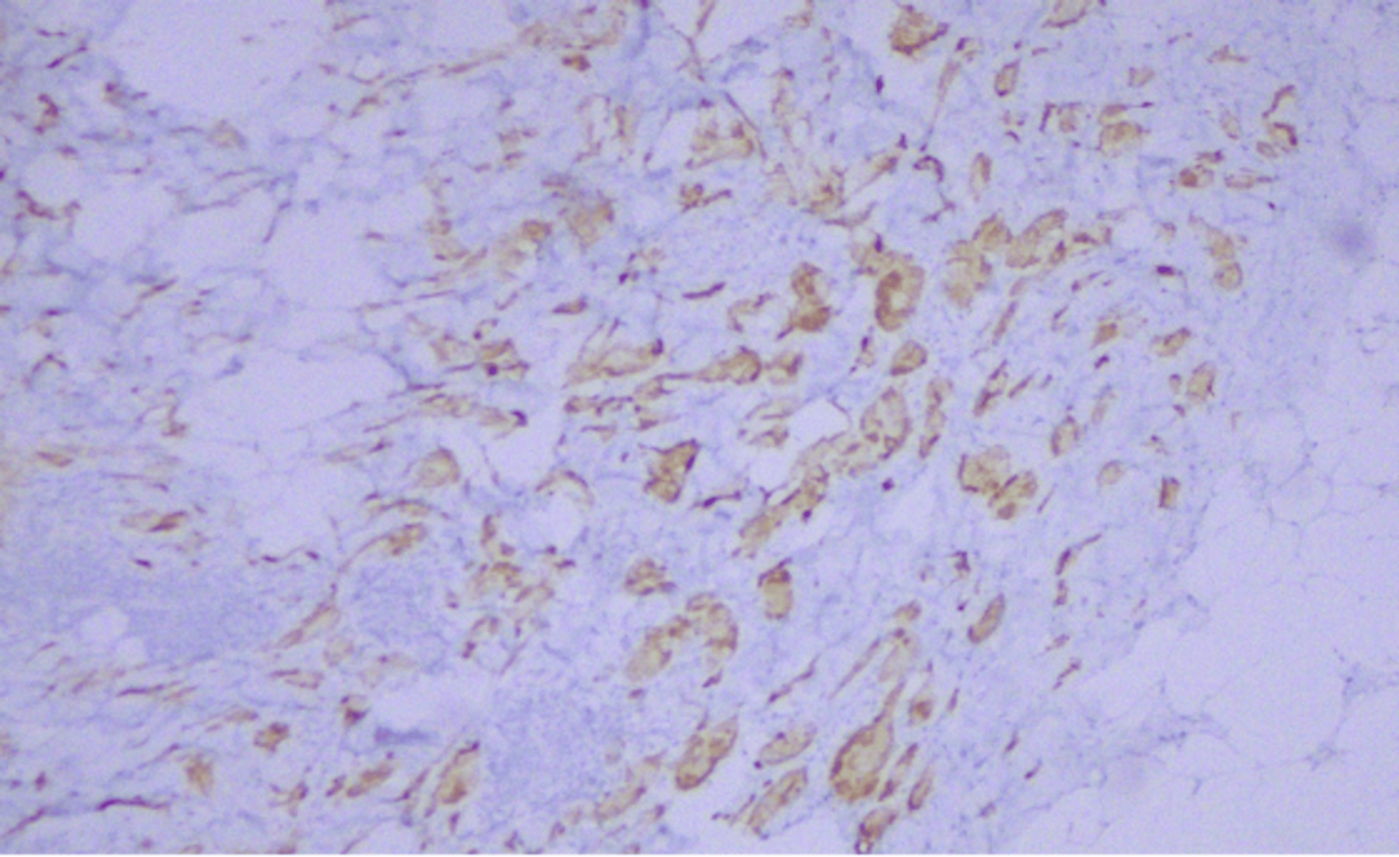
Immunohistochemistry: inhibin positivity.

Immunohistochemical analysis of the lesion cells demonstrated diffuse expression of S-100, CD68, inhibin, and SOX10, and negativity for CK AE1/AE3, GATA3, MART-1, and HMB-45 (Figures 5-7). The Ki-67 proliferation index was extremely low (<1%).

The lesion appeared to be confined within the breast parenchyma, without extension to the peripheral/lateral or deep resection margins. The excised skin segment was free of pathological involvement. The remaining breast parenchyma shows diffuse atrophy of the terminal duct-lobular units and scattered areas of fibrosis.

Taking into account the above morphological and immunohistochemical findings, we conclude that this represents a GCT of the breast, not otherwise specified, with benign biological behavior. The patient remains disease-free to date, with 1.5 years of follow-up.

## Discussion

Granular cell tumors (GCTs) are rare neoplasms that may arise in various anatomical sites, most frequently within the oral cavity, particularly the tongue. Breast involvement is uncommon [3,16]. Although their histogenesis remains debated, current evidence supports a Schwann cell origin [2,3].

Breast GCTs have been reported across a broad age range (15-74 years) and in both sexes; however, they predominantly affect premenopausal women, with a mean age of approximately 32 years, and show a slight predominance in individuals of African descent [18,19]. In contrast, our patient was a postmenopausal woman, representing a less typical demographic presentation.

Clinically and radiologically, these lesions often mimic breast carcinoma, posing a diagnostic challenge. They typically present as firm, painless masses and may be associated with skin retraction or tethering due to their superficial location [4]. Although most commonly identified in the upper inner quadrant, they may occur in other regions of the breast, occasionally appearing fixed to the pectoralis muscle and thus raising suspicion for malignancy [4]. In our case, the lesion was located in the upper outer quadrant, which is a common site for breast carcinoma, further increasing the potential for misdiagnosis. While generally solitary, multifocal disease has been described in a minority of cases.

Imaging findings are variable and non-specific. Lesions may appear well-circumscribed or ill-defined, particularly in the presence of reactive fibrosis, and can demonstrate regular, spiculated, or lobulated margins. Notably, calcifications are typically absent, a finding that was also observed in the present case [20]. 

Macroscopically, GCTs are firm, well-circumscribed masses with a homogeneous yellow-tan cut surface. In some cases, ill-defined borders and infiltration into adjacent structures, including the pectoralis muscle, have been described. Axillary lymphadenopathy is uncommon [4,10].

Definitive diagnosis relies on histopathological evaluation, usually obtained via core needle biopsy [10]. Microscopically, GCTs consist of polygonal cells arranged in nests or sheets, with abundant eosinophilic granular cytoplasm due to lysosomal accumulation. Nuclei are typically small, uniform, and centrally located. The absence of mitotic activity, cytologic atypia, pleomorphism, and necrosis supports their benign nature [2,4].

Malignant transformation is rare (1-2%). Tumors that meet three or more of the Fanburg-Smith criteria are considered malignant, while those meeting two criteria are classified as atypical [10,14]. Immunohistochemically, GCTs characteristically show strong and diffuse S100 positivity, supporting their neural origin, whereas CD68 may be variably expressed. Additional markers such as vimentin and inhibin have also been reported. Typically, these tumors are negative for epithelial markers and hormone receptors.

Importantly, our patient remains in excellent clinical condition following wide local excision with tumor-free margins, which represents the treatment of choice for these lesions. Complete surgical excision with negative margins is essential to prevent local recurrence.

In selected cases, adjunctive techniques, such as the use of SPIO nanoparticles (Magtrace), may be considered. When administered preoperatively, SPIO tracers can facilitate the identification of sentinel lymph nodes without the use of radioactive isotopes. Compared with conventional radiotracer-based methods, SPIO offers advantages including the absence of ionizing radiation, reduced reliance on nuclear medicine facilities, and greater flexibility in surgical scheduling. SPIO particles may also remain detectable in lymphatic tissue for an extended period following injection, in some cases for more than one month. This may be particularly useful in cases of diagnostic uncertainty or when malignancy cannot be definitively excluded preoperatively, allowing for more flexible surgical planning [17].

## Conclusions

This case highlights the diagnostic challenge posed by granular cell tumors of the breast, which can closely mimic malignancy both clinically and radiologically. Accurate diagnosis relies on careful radiologic-pathologic correlation and histopathological confirmation. Complete surgical excision with clear margins remains the treatment of choice and is associated with an excellent prognosis.

In selected cases with diagnostic uncertainty, adjunctive techniques, such as sentinel lymph node evaluation, may be considered. Emerging approaches, including the use of SPIO tracers, may offer additional flexibility in surgical planning, particularly by allowing preoperative localization without the need for radioactive isotopes. Overall, this case underscores the importance of a multidisciplinary approach to avoid misdiagnosis and overtreatment.
